# Anomalous effect of the aging degree on the ionic permeability of silica shells†

**DOI:** 10.1039/c8ra08936a

**Published:** 2018-11-15

**Authors:** Shenghua Wang, Chaoran Li, Zhijie Chen, Zhijie Zhu, Qishan Zhu, Ruijun Tang, Wei Sun, Le He, Xiaohong Zhang

**Affiliations:** Institute of Functional Nano & Soft Materials (FUNSOM), Jiangsu Key Laboratory for Carbon-Based Functional Materials & Devices, Soochow University 199 Ren'ai Road Suzhou 215123 Jiangsu P. R. China lehe@suda.edu.cn xiaohong_zhang@suda.edu.cn; Jiangsu Key Laboratory of Thin Films, College of Physics, Optoelectronics and Energy, Soochow University Suzhou 215006 Jiangsu P. R. China; Department of Chemistry, University of Toronto 80 St. George Street Toronto Ontario M5S 3H6 Canada wsun@chem.utoronto.ca

## Abstract

We present a systematic study on the ionic permeability and protective ability of silica shells with different aging degrees by using the acid etching of silica-coated iron oxide nanoparticles as the model reaction. Contradictory to common impressions, we found that the ionic permeability of silica shells increased rather than decreased with the increasing aging degree. This trend may be explained by the chemical nature of the sol–gel silica shell that affects the wettability and, thereby, the transportation of water molecules and hydrated ions. Our study provides novel insights into the protective ability of sol–gel derived silica, which enables us to design thin but low-permeability shells for the stability of inner nanoparticles under harsh conditions without scarifying the performance of core–shell nanostructures.

## Abstract 2

We discovered an anomalous effect of the aging degree on the ionic permeability of colloidal silica.

## Graphical abstract

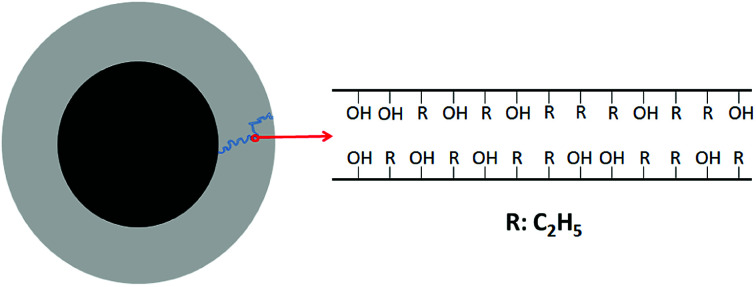

## 1. Introduction

Owing to their high specific surface areas, nanoparticles exhibit higher chemical reactivity, but lower stability than their bulk counterparts.^1–8^ Increasing the stability of nanoparticles, particularly under harsh conditions, is crucial for their widespread applications in different areas such as catalysis, photonics, environmental remediation, biomedicine, energy conversion and storage.^9–17^ For example, functionalized magnetite-based nanoparticles have been utilized as magnetically recyclable adsorbents for the removal of heavy metal ions from acidic waste water.^18–20^ Nevertheless, they often suffer from the corrosion of iron oxide in acidic environments, leading to the decreased recycling efficiency through magnetic separation and the unwanted loss of adsorbents. In this context, surface coating with a layer of sol–gel silica to construct core–shell structures has been used as a general method to improve the stability of the inner nanoparticles.^21–28^ For example, Tong *et al.* reported the use of poly(1-vinylimidazole)-grafted Fe_3_O_4_@SiO_2_ particles with improved stability as reusable adsorbents for the removal of Hg^2+^ in waste water.^25^

The protective ability of silica shells depends on their permeability to species that attack the inner nanoparticles. Therefore, it is usually necessary to decrease the shell permeability in the core–shell nanostructures for more effective stabilization under harsh conditions. Conventionally, this requires increasing the thickness of silica shells, which often leads to a decreased level of performance of the inner nanoparticles.^29–32^ For instance, Dravid *et al.* reported that the magnetic resonance imaging (MRI) performance of Fe_3_O_4_@SiO_*x*_ particles decreased with the increase of the silica shell thickness.^30^ Chu *et al.* found that the photoluminescence of silica-coated lanthanide complexes weakened when increasing the shell thickness.^31^ The use of thick shells also increases the overall particle size and, thus, greatly limits the bio-related applications of silica-coated nanoparticles.^16,33–35^ Therefore, it is of emerging interest to design thin but low-permeability silica shells towards the stabilization of nanoparticles.

Herein we present a systematic study on the protective ability of silica shells with different aging degrees by using the acid etching of silica-coated iron oxide nanoparticles as the model reaction. Our study provides substantial evidences that the permeability of silica shells strongly depends on their aging degrees. Surprisingly we observed that less aged silica shells exhibit lower ionic permeability, and thus better protective ability. This may be explained by the contrast in the wettability between silica shells with different aging degrees. Our study provides novel insights into the protective ability of sol–gel derived silica. This knowledge enables us to design thin but low-permeability shells for the stability of inner nanoparticles under harsh conditions without sacrificing the performance of core–shell nanostructures.

## 2. Experimental section

### 2.1. Materials

All the chemicals were used as received without further purification. Polyvinylpyrrolidone (PVP, *M*_w_ = 360 000), monosodium dihydrogen orthophosphate (NaH_2_PO_4_, reagent grade) were obtained from Vetec. Ferric chloride hexahydrate (FeCl_3_·6H_2_O, reagent grade) and PSSMA [poly(4-styrenesulfonic acid-co-maleic acid) sodium salt] were purchased from Sigma Aldrich. Sodium acetate anhydrous (99%), ethylene glycol (99.5%), tetraethyl orthosilicate (TEOS, >96%), ammonium hydroxide solution (NH_3_·H_2_O, 28 wt%), ethanol (GR, ≥99.8%) and hydrochloric acid (36–38%, analytical reagent) were purchased from Energy chemical, J&K scientific, TCI, Macklin, Sinopharm Chemical Reagent Co., Ltd and Enox, respectively. Milli-Q water (Millipore, 18.2 MΩ cm at 25 °C) was used in all experiments.

### 2.2. Characterization

Transmission electron microscopy (TEM) images were obtained with an FEI-Tecnai F20 (200 kV) transmission electron microscope. The particle size distribution of different samples was counted *via* Nano Measurement (at least 100 particles were included for each sample). Fourier transform infrared (FTIR) spectra were obtained with an FTIR spectrometer (Spectrum One, PerkinElmer) using a standard KBr pellet technique. The Brunauer–Emmett–Teller (BET) data were collected from a Micromeritics ASAP 2020 HD88. The Fe content of different samples was measured by an inductively coupled plasma source mass spectrometer (ICP-MS) (Aurora M90, Jenoptik). The magnetic properties were measured using a vibrating sample magnetometer (VSM) in the physical property measurement system (PPMS, Quantum Design).

### 2.3. Synthesis of Fe_3_O_4_ colloidal nanocrystal clusters (CNCs)

Fe_3_O_4_ CNCs were synthesized according to a reported recipe.^36^ Under magnetic stirring, 7.5 g of PSSMA was dissolved in ethylene glycol (300 mL) to form a clear solution, followed by the addition of FeCl_3_·6H_2_O (8.1 g) and sodium acetate (22.5 g). The obtained homogeneous red brown solution was then sealed in a Teflon-lined stainless-steel autoclave and heated at 200 °C for 10 hours. When cooled down, the dark precipitates were isolated by a magnet and washed 6 times with Milli-Q water and ethanol alternately, and finally dispersed in ethanol to form a suspension with a concentration of 10 mg mL^−1^.

### 2.4. Synthesis of α-Fe_2_O_3_ ellipsoidal particles

Ellipsoidal α-Fe_2_O_3_ particles were synthesized according to a reported recipe.^37^ 1.3 g of Fe_2_Cl_3_·6H_2_O, 80 μL of NaH_2_PO_4_ solution (0.1 mg μL^−1^), and 400 mL of Milli-Q water were mixed together under sonication. The mixture was then kept in an oven at 100 °C for 48 hours. The product was collected by centrifugation and washed with Milli-Q water several times, which was finally dispersed in certain amount of Milli-Q water to form a suspension with a concentration of 10 mg mL. 5 mL of the suspension was diluted by 25 mL of Milli-Q water, followed by the addition of 5 mL of PVP aqueous solution (*M*_w_: 360 000, 0.04 g mL^−1^). The mixture was then stirred overnight. The PVP modified α-Fe_3_O_4_ particles was collected by centrifugation at 11 000 rpm for 30 min. The supernatant was discarded and the precipitate was redispersed in certain amount of ethanol to form a suspension with a concentration of 10 mg mL^−1^.

### 2.5. SiO_2_ coating

The Fe_3_O_4_ CNCs and PVP-modified α-Fe_2_O_3_ nanoparticles were then coated with a thin layer of silica *via* a modified sol–gel method.^38^ Briefly, 20 mL of the Fe_3_O_4_ CNCs or α-Fe_2_O_3_ suspension was diluted by 60 mL of ethanol, followed by the addition of 12 mL of Milli-Q water. The mixture was then sonicated for 30 minutes. 10 mL of ammonium hydroxide (28%) aqueous solution and a certain amount of TEOS (500 μL for CNCs; 700 μL for α-Fe_2_O_3_) were added into the suspension sequentially. The reaction container was then transferred to a shaking bed, sampled after a certain period. The products were obtained by centrifugation and washed twice with ethanol. Finally, they were dispersed in ethanol with a concentration of 10 mg mL^−1^.

### 2.6. Acid etching

5 mL of the suspension of Fe_3_O_4_@SiO_2_ particles (with different sol–gel reaction times) was centrifuged at 13 000 rpm for 6 min. The precipitate was redispersed in 10 mL of 1 M HCl. The reaction container was then transferred to a shaking bed (400 rpm, 20 °C), sampled after a certain time. The sample was centrifuged immediately at 13 000 rpm for 6 min. The Fe content of the supernatant was detected *via* Inductively coupled plasma source mass spectrometer (ICP-MS). Part of the precipitate was redispersed in ethanol for TEM characterization and the remains was dried (100 °C, 3 h, in an oven) for magnetism analysis. 2.5 mL of the suspension of α-Fe_2_O_3_@SiO_2_ (with different sol–gel reaction time) was centrifuged at 11 000 rpm for 10 min. The supernatant was discarded and the precipitate was redispersed in 15 mL of 8.33 M HCl. The reaction container was then transferred to a shaking bed (400 rpm, 30 °C), sampled after 1.5 h. The sample was centrifuged immediately at 13 000 rpm for 6 min. The supernatant was kept for content analysis of Fe *via* ICP-MS. The precipitation was re-dispersed in ethanol for TEM characterization.

### 2.7. Cycling stability of Fe_3_O_4_ in adsorption of Cu^2+^

The Fe_3_O_4_@SiO_2_ particles were first functionalized with amino groups according to a reported recipe.^39^ N^1^-(3-Trimethoxysilylpropyl) diethylenetriamine (0.5 mL) and *N*,*N*-diisopropylethylamine (0.1 mL) were added to the mixture of Fe_3_O_4_@SiO_2_ nanoparticles (100 mg) and ethanol (40 mL). After stirring for 12 h, the amino-modified nanoparticles were collected by centrifugation, cleaned with ethanol for several times, and dispersed in water. The cycling stability of Fe_3_O_4_ cores in the adsorption of Cu^2+^ was then tested. In a typical adsorption–desorption cycle, 100 mg of the amino-modified particles were mixed with 5 mL of aqueous Cu^2+^ solution (0.005 M, pH = 5), followed by magnetic stirring for 1.5 h. After the adsorption, the magnetic particles were collected from the dispersion through magnetic separation. For the desorption of Cu^2+^, the recovered particles were then redispersed in 5 mL of 1 M HCl, followed by magnetic stirring for 1.5 h. Finally, the magnetic particles were collected through magnetic separation and cleaned with water to removes all copper ions and regenerate the adsorption sites.

## 3. Results and discussion

Stöber method has been proven as an effective route to coat nanoparticles with silica shells.^40,41^ The sol–gel process involves the hydrolysis and condensation of a silane precursor (*e.g.* tetraethyl orthosilicate).^42^ Base catalysts (*e.g.* ammonium hydroxide) will accelerate the hydrolysis of the Si–OR (R = CH_2_CH_3_ for TEOS) groups to form silanol groups (Si–OH). The further condensation of these silanol bonds will lead to the formation of Si–O–Si groups. However, the hydrolysis and condensation are imperfect, and unhydrolysed Si–OR and uncondensed Si–OH groups exist inside newly formed silica shells.^43–45^ Therefore, sol–gel silica colloids contain three functional groups: Si–O–Si, Si–OH and Si–OR. The amounts of Si-OR groups and Si–O–Si groups determine the hydrolysis and condensation degrees, respectively. As the sol–gel reaction time (aging time) prolongs, the hydrolysis and condensation degrees (or aging degree) increase for silica shells.^41^ Our hypothesis is that the aging degree of silica shells may influence the ionic permeability and, thereby, protective ability.

**Fig. 1:**
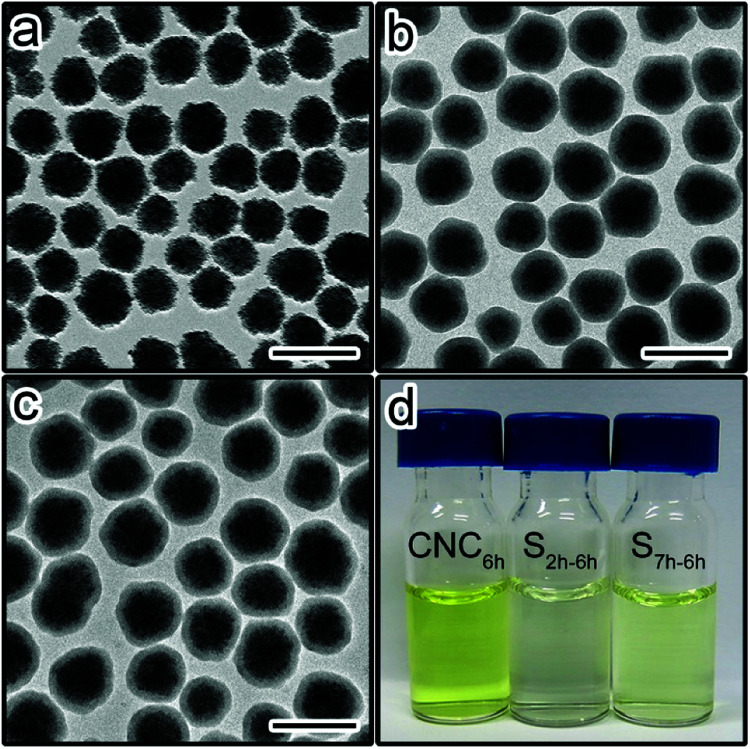
TEM image of (a) Fe_3_O_4_ CNCs, (b) CNCs@SiO_2_ particles with the sol–gel reaction time of 2 hours (sample S_2 h_), and (c) CNCs@SiO_2_ particles with the sol–gel reaction time of 7 hours (sample S_7 h_). Scale bars are 200 nm. (d) Photograph of the supernatants of different etched samples.

To verify our hypothesis, an acid etching experiment was conducted on Fe_3_O_4_@SiO_2_ nanoparticles to investigate the relationship between the aging time and the permeability of silica shells. Fe_3_O_4_ CNCs particles with an average diameter of 113 nm were prepared and encapsulated with a thin layer of sol–gel silica. Fig. 1a shows the transmission electron microscopy (TEM) image of the Fe_3_O_4_ particles, denoted as CNC. We also prepared two Fe_3_O_4_@SiO_2_ samples with the sol–gel reaction time of 2 hours and 7 hours (denoted as S_2 h_ and S_7 h_), respectively. As shown in Fig. 1b and c, both samples have the same average shell thickness of 20 nm. Thus, the influence of the shell thickness on the permeability can be neglected.

The same amount of CNCs, S_2 h_ and S_7 h_ were etched by HCl (1 M) for 6 hours. The samples were centrifuged immediately after the etching. Fig. 1d shows the digital photos of the supernatants of the etched samples (denoted as CNC_6h_, S_2 h-6 h_, and S_7 h-6 h_, corresponding to the original samples CNCs, S_2 h_ and S_7 h_, respectively). The etching of Fe_3_O_4_ was directly evidenced by the yellow colour of the supernatants. The supernatant of CNC_6h_ appeared the darkest while the colour is the lightest for S_2 h-6 h_. ICP-MS results confirmed the decrease of the Fe content in the supernatants from CNC_6h_ to S_7 h-6 h_, and then to S_2 h-6 h_ (Fig. S1†). These results suggest the etching rate of Fe_3_O_4_ is the fastest for CNCs without the silica protection. More importantly, the etching rate increased from S_2 h-6 h_ to S_7 h-6 h_, suggesting the shell permeability was different for the two samples. Since the two samples were made of the same CNCs cores with the same thickness of silica shells, it is most likely that the difference in the permeability could be attributed to the aging degree of silica shells.

**Fig. 2:**
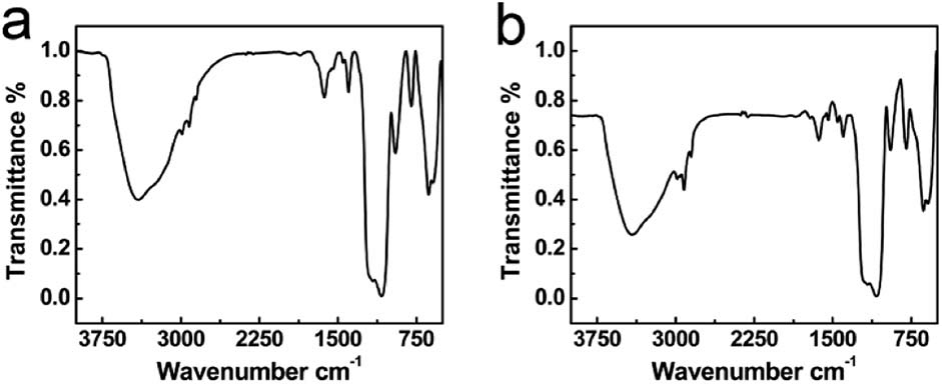
Normalized FTIR spectra of (a) S_2 h_ and (b) S_7 h_.

Fourier transform infrared spectroscopy (FT-IR) was used to investigate the aging degrees of silica shells in S_2 h_ and S_7 h_. Fig. 2a and b depicts the normalized FT-IR spectra of sample S_2 h_ and S_7 h_, respectively. The difference in the hydrolysis and condensation degrees between the two samples was revealed from the peak intensity at 2980 cm^−1^ and 1050 cm^−1^, which could be assigned to the Si–O–R and Si–O–Si groups, respectively.^46,47^ To better compare their aging degrees, we performed a quantitative calculation similar to our previous study.^41^ The peak area ratio of the Si–O–R group (2980 cm^−1^) to the sum of Si–O–Si (1050 cm^−1^) and Si–OH (960 cm^−1^) groups, R_1_, was used to compare the relative hydrolysis degree. The peak area ratio of the sum of Si–O–R and Si–OH groups to the Si–O–Si group, R_2_, was used to compare the relative condensation degree. The values of R_1_ and R_2_ of the two samples were listed in Table 1. Both R_1_ and R_2_ decreased from S_2 h_ to S_7 h_, confirming a higher aging degree for S_7 h_ with a longer sol–gel reaction time.^41^ These results suggest that the permeability of silica shells increases with the aging degree, which seems controversial to the common impression that more condensed gels are less permeable.

**Table 1:** The comparison of the IR peak areas corresponding to different functional groups for different samples

Sample	*t* a (h)	R_1_^b^	R_2_^c^
S_2 h_	2	0.3678	0.5003
S_7 h_	7	0.2548	0.3094

a The initial sol–gel reaction time for silica coating.

b The peak area ratio of the Si–O–R group (2980 cm^−1^) to the sum of Si–O–Si (1050 cm^−1^) and Si–OH (960 cm^−1^) groups. The lower of this ratio, the higher the hydrolysis degree.

c The peak area ratio of the sum of Si–O–R and Si–OH groups to the Si–O–Si group. The lower of this ratio, the higher the condensation degree.

**Fig. 3:**
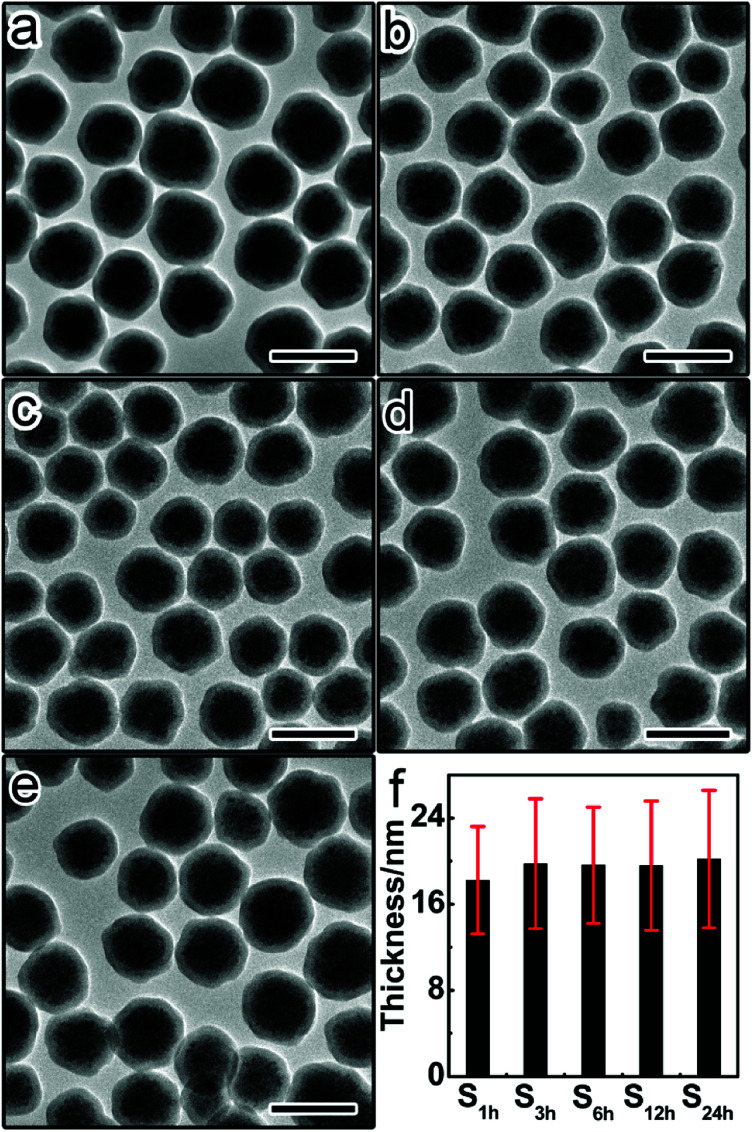
TEM image of CNCs@SiO_2_ particles with the sol–gel reaction time of (a) 1 hour (sample S_1 h_), (b) 3 hours (sample S_3 h_), (d) 6 hours (sample S_6 h_), (d) 12 hours (sample S_12 h_), and (e) 24 hours (sample S_24 h_). Scale bars are 200 nm. (f) Distributions of the shell thickness for different samples.

To thoroughly investigate the dependence of permeability on the aging degree of silica shells, we further prepared a set of Fe_3_O_4_@SiO_2_ particles with similar thicknesses but different aging degrees (Fig. 3). The sol–gel reaction time of these samples (denoted as S_1 h_, S_3 h_, S_6 h_, S_12 h_, and S_24 h_, respectively) was varied between 1 hour, 3 hours, 6 hours, 12 hours, and 24 hours. As the sol–gel reaction time prolongs, the aging degree increases continuously from S_1 h_ to S_24 h_.^41^ The core–shell structure remains intact regardless of the aging time (Fig. 3a–e). As shown in Fig. 3f, the shell thickness was almost the same between this set of samples, which is beneficial for investigating the effect of aging degree on the permeability of silica shells.

**Fig. 4:**
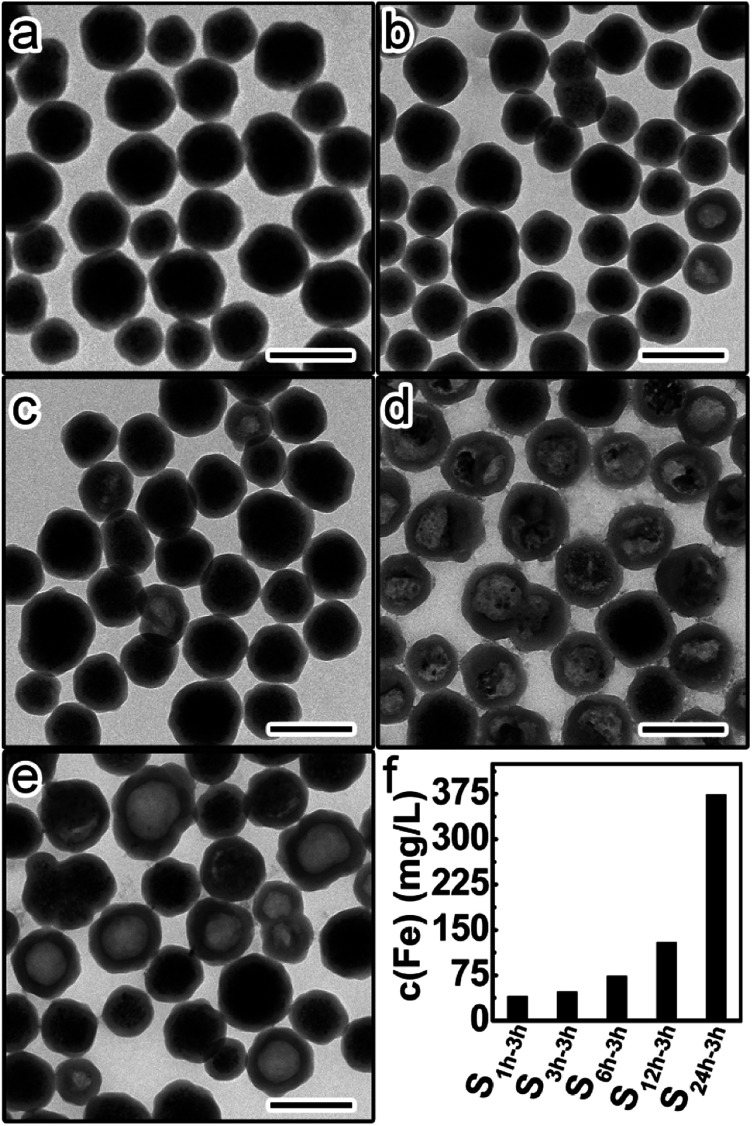
TEM images of the precipitates from different etched samples: (a) S_1 h-3 h_, (b) S_3 h-3 h_, (c) S_6 h-3 h_, (d) S_12 h-3 h_, and (e) S_24 h-3 h_. Scale bars are 200 nm. (f) Fe contents in the supernatants of different etched samples.

This set of five samples were etched by HCl (1 M) for 3 hours, followed by immediate centrifugation. Fig. 4a–e shows the TEM images of the precipitates from different etched samples. The amount of residual Fe_3_O_4_ in the precipitates was found to continuously decrease from S_1 h-3 h_ to S_24 h-3 h_, suggesting the increase of the etching rate with higher aging degrees of silica shells. This trend was further confirmed by the gradual increase of the Fe content in the supernatants from S_1 h-3 h_ to S_24 h-3 h_ (Fig. 4f). These results prove that silica shells become more permeable to HCl and less protective when increasing the aging degree.

Such discrepancy in the protective ability was even more significant if we extended the etching time to 10 hours. A similar trend in the amount of remaining Fe_3_O_4_ was found in the etched samples (Fig. S2a–e†). The difference in the Fe content of the supernatant is much more pronounced between S_1 h-10 h_ and any of the other four etched samples (Fig. S2f†). These results further demonstrate that the protective ability of silica shells is strongly dependent on their aging degrees.

**Fig. 5:**
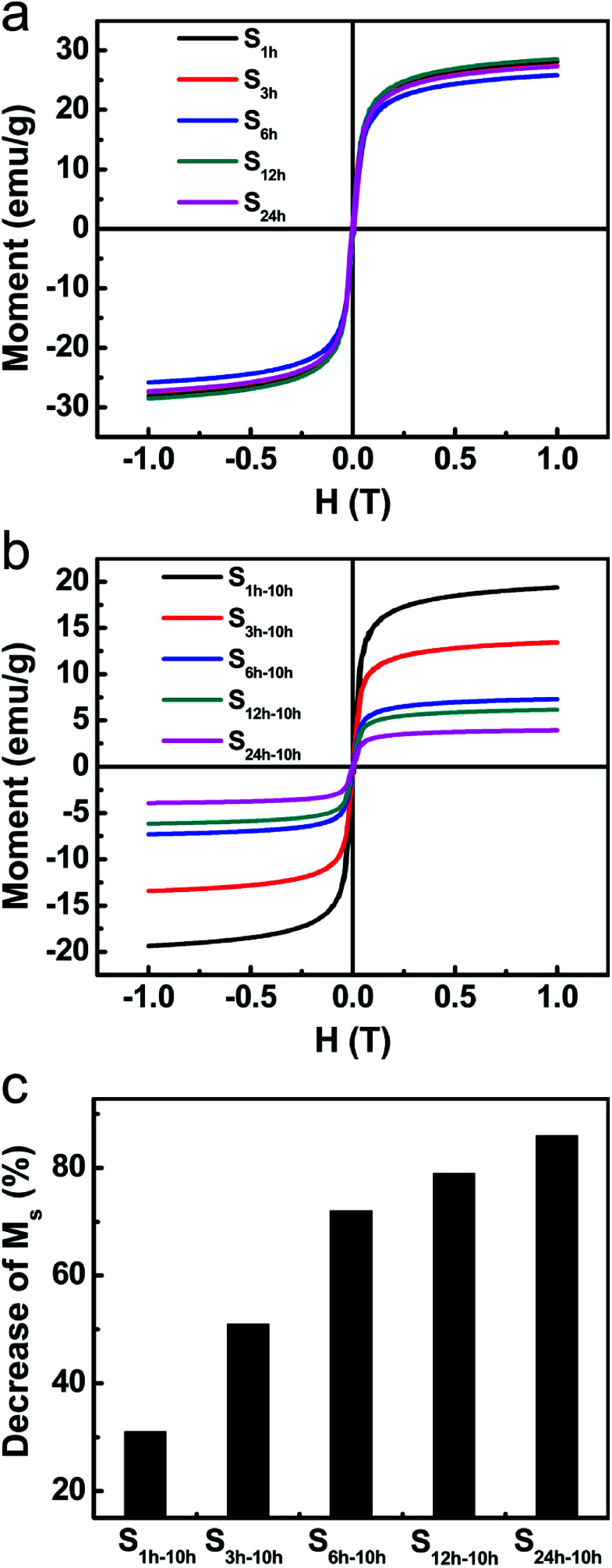
(a) The magnetic hysteresis loops of S_1 h_, S_3 h_, S_6 h_, S_12 h_, and S_24 h_ at room temperature. (b) The magnetic hysteresis loops of precipitates of S_1 h-10 h_, S_3 h-10 h_, S_6 h-10 h_, S_12 h-10 h_, and S_24 h-10 h_ at room temperature. (c) Relative drop of the saturation magnetization after etching.

The higher etching rate of Fe_3_O_4_ for samples with more aged silica shells was also evidenced by the magnetization measurements. Fig. 5a shows the hysteresis loops of the above-mentioned five samples before etching. All samples were superparamagnetic at room temperature with negligible coercive forces (*H*_c_) and remanent magnetizations (*M*_r_). The saturation magnetizations were almost the same for this set of five samples. The slight difference resulted from the small variance in the shell thickness. After the 10 hour etching, we observed an obvious and continuous drop in the saturation magnetization (*M*_s_) from S_1 h-10 h_ to S_24 h-10 h_ (Fig. 5b and c). This trend is consistent with the fact that more amounts of Fe_3_O_4_ were etched away for samples with more aged silica shells.

As shown above, we found that silica shells with higher aging degrees are more permeable to ions. One plausible explanation for this observed trend is that the wettability of silica shells is affected by their aging degrees. Compared with the Si–OC_2_H_5_ group, the Si–OH group exhibits stronger hydrogen bonding ability with water and is, thus, more hydrophilic. The presence of more hydrophobic Si–OC_2_H_5_ groups in less aged silica shells, as confirmed by FT-IR studies, hinders the transportation of water molecules and hydrated ions (such as H^+^, Fe^2+^ and Fe^3+^). In contrast, the ionic permeability is greatly improved for more aged silica shells with a lower number of Si–OC_2_H_5_ groups but a higher concentration of hydrophilic Si–OH groups. One may propose another possibility that the aging process may create pores that serve as the ionic channels.^32^ However, no micropores or mesopores were found in either S_1 h_ or S_24 h_ from the Brunauer–Emmett–Teller (BET) measurements (Fig. S3†). It is most likely that the contrast in the wettability accounts for the observed trend. While further studies are necessary to fully elucidate the mechanism, the present study clearly demonstrates that the protective ability of silica shells can be chemically tuned by simply varying the aging degree.

Besides Fe_3_O_4_ CNCs, we further studied the etching of silica-coated α-Fe_2_O_3_ nanoparticles in an attempt to justify the versatility of the aging degree effect. Ellipsoidal α-Fe_2_O_3_ nanoparticles were coated with a thin layer of silica to prepare two α-Fe_2_O_3_@SiO_2_ samples with the same shell thickness (Fig. S4a and b†). The sol–gel reaction time was varied between 1 hour and 24 hours. After being treated in HCl under the same conditions, more α-Fe_2_O_3_ were etched away for the 24 hour sample (Fig. S4c–f†). These results again suggest that silica shells with higher aging degree exhibit higher ionic permeability regardless of the core composition and particle shape.

**Fig. 6:**
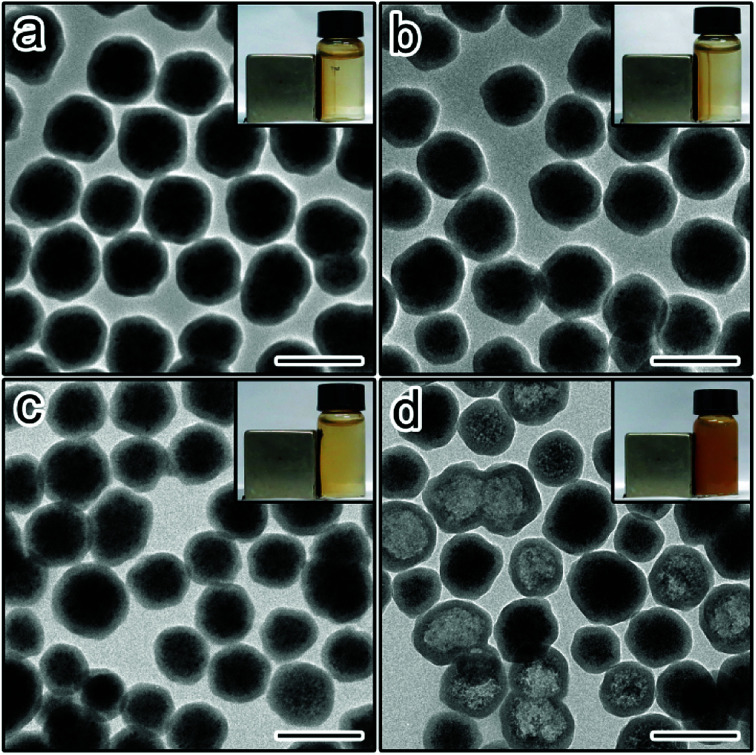
TEM image of (a) S_1 h_–NH_2_, (b) S_24 h_–NH_2_, (c) S_1 h_–NH_2_ after 8 cycles of Cu^2+^ removal experiments, and (d) S_2 4h_–NH_2_ after 8 cycles of Cu^2+^ removal experiments. Scale bars are 200 nm. The inset in each image shows the corresponding samples dispersed in water after magnetic separation for 60 s.

Thanks to the improved understanding of the aging degree effect, it is now possible to rationally tune the protective ability of silica shells without changing the thickness, *e.g.* simply by adjusting the sol–gel reaction time. Finally, we demonstrate the use of thin but low-permeability shells to improve the cycling stability of iron oxide nanoparticles when used in the removal of metal ions. Notably, the desorption of metal ions is usually carried out under strongly acidic conditions (*e.g.* 1 M HCl). This raises the concern on the etching of iron oxide and the decrease of the recycling efficiency of nanoparticles through magnetic separation. Two samples S_1 h_ and S_24_ were functionalized with amino groups. The modified samples (denoted as S_1 h_–NH_2_ and S_24 h_–NH_2_, respectively) were then used as the adsorbents in the removal of Cu^2+^. Magnetic separation was used for the recovering and cleaning of the particles. In the first cycle, both magnetic nanoparticles could be quickly separated from the dispersion within 60 seconds (Fig. 6a and b inset). TEM images revealed that the core–shell structures remained intact after the first cycle (Fig. 6a and b). However, the difference between the two samples appeared gradually during the cycling tests. For example, in the 8^th^ cycle, no obvious drop in the separation efficiency was found for S_1 h_–NH_2_ (Fig. 6c inset). This is consistent with the preservation of the Fe_3_O_4_ cores after 8 cycles (Fig. 6c). In contrast, severe etching of Fe_3_O_4_ occurred for S_24 h_–NH_2_ (Fig. 6d). Consequently, the magnetic separation process became much slower, leading to unwanted losses of adsorbents (Fig. 6d inset). It is believed that these thin but low-permeability silica shells can also stabilize nanoparticles with different compositions and functionalities, facilitating their applications in different areas.

## 4. Conclusions

In summary, we have systematically studied the effect of the aging degree on the ionic permeability of silica shells. Contradictory to common impressions, we found that the ionic permeability of silica shells increased rather than decreased with the increasing aging degree. This trend may be explained by the chemical nature of the silica shell that affects the wettability, and thereby the transportation of water molecules and hydrated ions. More importantly, our study provides a feasible and effective way for chemically improving the protective ability of silica shells by simply controlling the sol–gel reaction time, which does not rely on the increase of the shell thickness that usually scarifies the performance of nanoparticles. Such insights into the protective ability of sol–gel derived silica would facilitate the practical implementation of silica-coated nanoparticles in the field of environmental remediation, demonstrated by a model experiment for repetitive Cu ion removal from water, as well as other potential applications in photonics, catalysis, and biomedicine.

## Conflicts of interest

There are no conflicts to declare.

## Supplementary Material

RA-008-C8RA08936A-s001
