# Supplementary material for: Anomalous effect of the aging degree on the ionic permeability of silica shells
Source: RSC Adv. 2018 Nov 15;8(67):38499–505. doi: 10.1039/c8ra08936a (PMC9090573; doi:10.1039/c8ra08936a)
Supplement: RA-008-C8RA08936A-s001 [file RA-008-C8RA08936A-s001.pdf]

Electronic Supplementary Material (ESI) for RSC Advances.
This journal is © The Royal Society of Chemistry 2018

Supplementary Information

Anomalous effect of the aging degree on the ionic permeability of silica shells

Shenghua Wang[1], Chaoran Li[1], Zhijie Chen[1], Zhijie Zhu[1], Qishan Zhu[2], Ruijun Tang[2], Wei Sun[3]*, Le He [1]*, and Xiaohong Zhang[1]*

[1]Institute of Functional Nano & Soft Materials (FUNSOM), Jiangsu Key Laboratory for Carbon-Based Functional Materials & Devices, Soochow University, 199 Ren'ai Road, Suzhou, 215123, Jiangsu, PR China.

Email: lehe@suda.edu.cn; xiaohong_zhang@suda.edu.cn

[2]Jiangsu Key Laboratory of Thin Films, College of Physics, Optoelectronics and Energy, Soochow University, Suzhou, 215006, Jiangsu, PR China.

[3]Department of Chemistry, University of Toronto, 80 St. George Street, Toronto, Ontario, M5S 3H6, Canada.

Email: wsun@chem.utoronto.ca

Supplementary Information

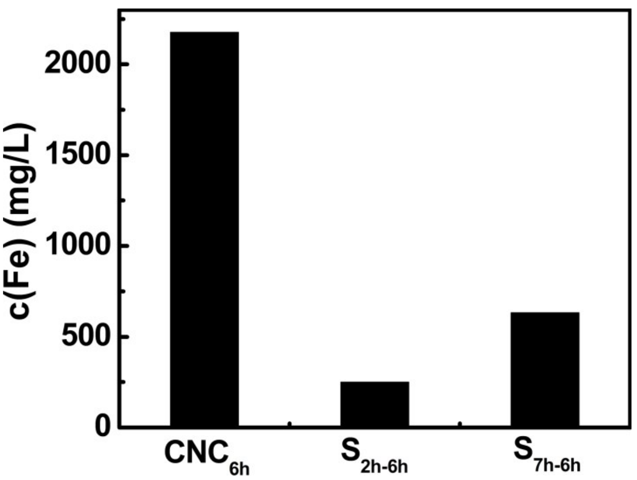

Figure S1. *ICP-MS results showing the decrease of the Fe content in the supernatants from* CNC_{6h} *to* $S_{7h\text{-}6h}$*, and then to* $S_{2h\text{-}6h}$

Supplementary Information

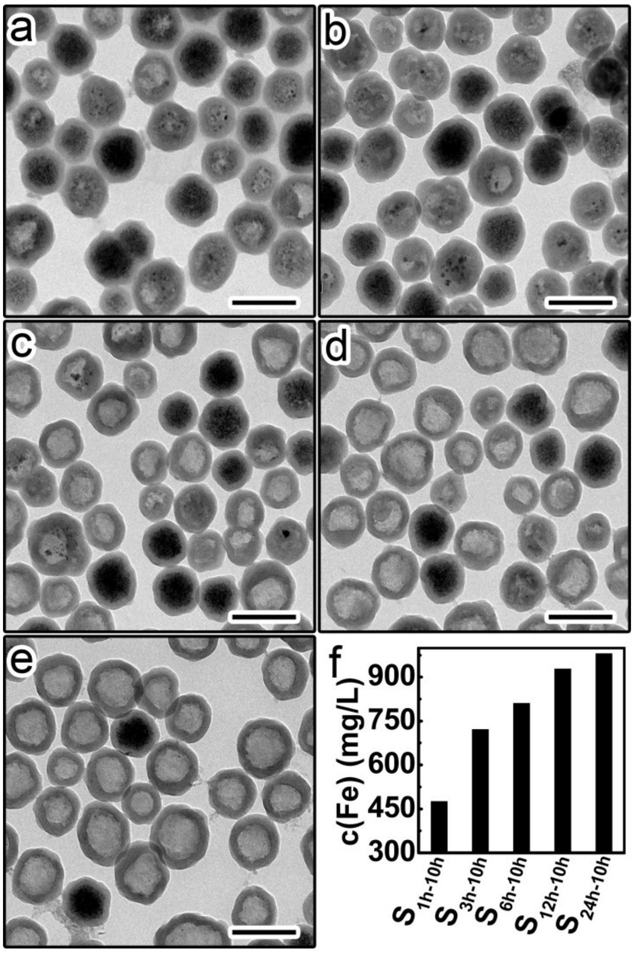

Figure S2. *TEM image of precipitates from different etched samples: (a) $S_{1h\text{-}10h}$, (b) $S_{3h\text{-}10h}$, (c) $S_{6h\text{-}10h}$, (d) $S_{12h\text{-}10h}$, and (e) $S_{24h\text{-}10h}$. Scale bars are 200 nm. (f) Fe contents in the supernatants of different etched samples.*

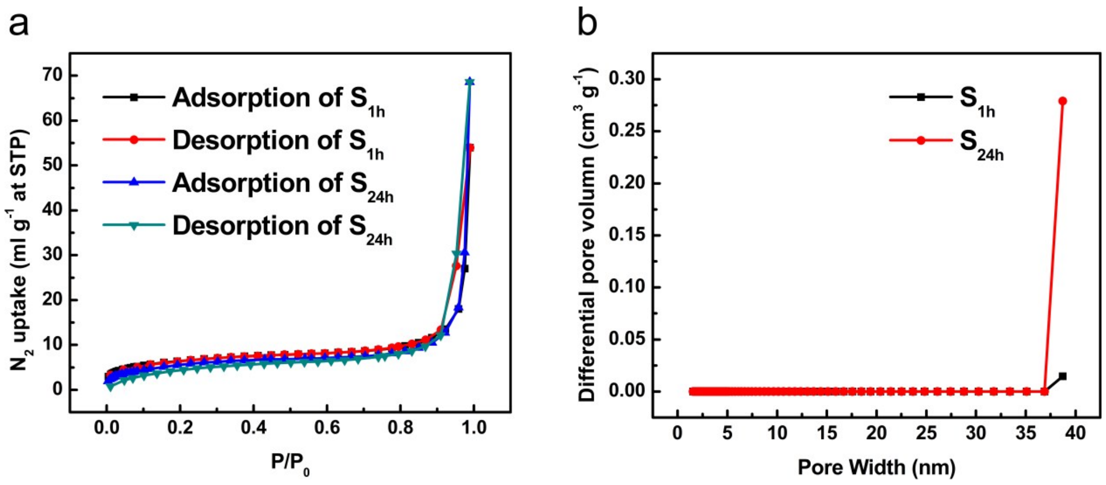

Figure S3. *(a) N_2 adsorption-desorption isotherms and (b) the corresponding pore size distributions of sample S_{1h} and S_{24h}.*

Supplementary Information

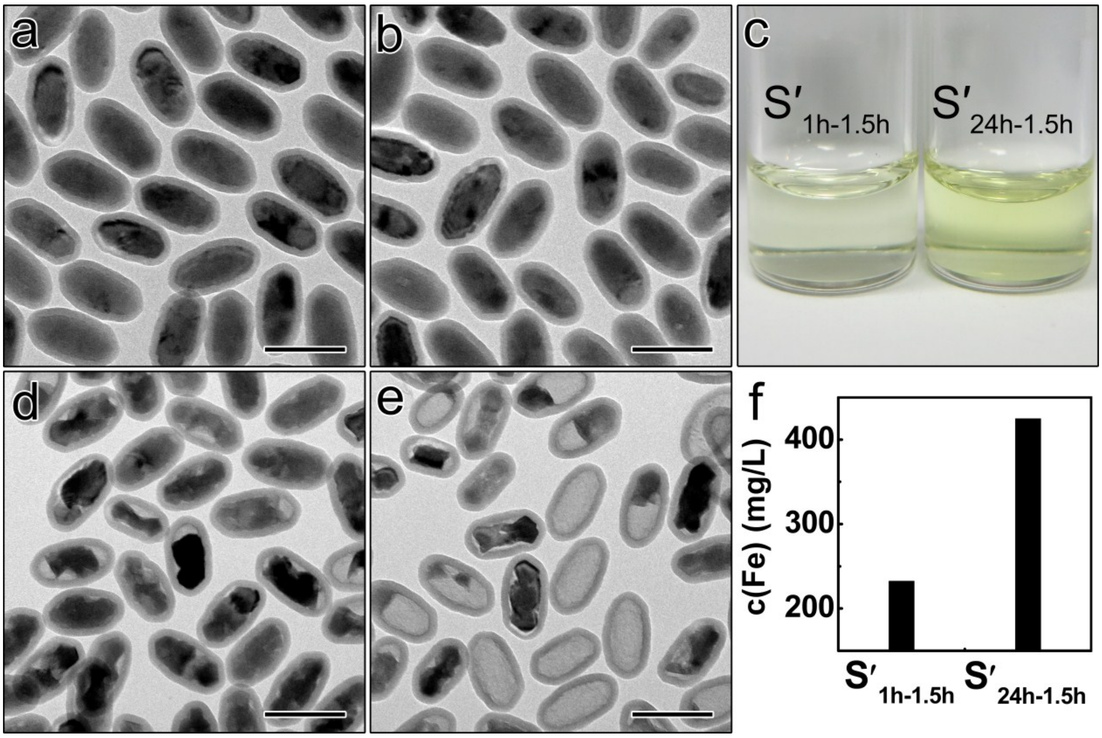

Figure S4. *TEM images of (a-b) α-Fe_2O_3@SiO_2 with the sol-gel reaction time of 1 hour (sample S'_{1h}) and 24 hours (sample S'_{24h}). (c) Optical photograph of the supernatant obtained by etching S'_{1h} and S'_{24h} (from left to right) for 1.5 hours, respectively. TEM images of precipitates of (d-e) $S'_{1h\text{-}1.5h}$ and $S'_{24h\text{-}1.5h}$. Scale bars are 200 nm. (f) Fe contents in the supernatants of S'_{1h} and S'_{24h} etched by 8.33 M HCl for 1.5 h.*
